# The Effect of Metalloestrogens on the Effectiveness of Aromatase Inhibitors in a Hormone-Dependent Breast Cancer Cell Model

**DOI:** 10.3390/cancers15020457

**Published:** 2023-01-11

**Authors:** Kamila Boszkiewicz, Helena Moreira, Ewa Sawicka, Anna Szyjka, Agnieszka Piwowar

**Affiliations:** 1 Department of Toxicology, Wroclaw Medical University, Borowska Street 211, 50-556 Wroclaw, Poland; 2 Department of Basic Medical Sciences, Faculty of Pharmacy, Wroclaw Medical University, Borowska Street 211, 50-556 Wroclaw, Poland

**Keywords:** breast cancer, xenoestrogens, aromatase inhibitors, metalloestrogens, chromium (III), aluminum, interaction

## Abstract

**Simple Summary:**

Progressive industrialization, urbanization, and consumerism lead to increased contamination of the environment with endocrine-disrupting compounds (EDCs) which play an important role in the increased incidence of hormone-dependent cancers, e.g., breast cancer. EDCs include, among others, xenoestrogens—exogenous compounds that can bind to estrogen receptors and thus compete with, or mimic the action of endogenous estrogens (e.g., promote the proliferation of cancer cells). The aim of the study was to answer the question whether exposure to selected xenoestrogens, widespread in everyday life (aluminum in antiperspirants and deodorants; chromium (III) in dietary supplements and drugs) affects the effectiveness of drugs used in hormone therapy in breast cancer. We performed in vitro tests on a breast cancer cell model—MCF-7 and MCF-7/DOX cell lines exposed to selected xenoestrogens, drugs, and their combinations. Our results confirm that such exposure may negatively affect the effectiveness of breast cancer hormone therapy.

**Abstract:**

Endocrine-disrupting compounds (EDC) play an important role in the increased incidence of breast cancer (BC). There are some 160 xenoestrogens that may be involved in the development of BC. Much less is known about the influence of xenoestrogens on the effectiveness of the treatment of BC. The aim of this study was to analyze the interaction of metalloestrogens (aluminum and chromium (III)) and drugs used in the treatment of hormone-dependent BC—aromatase inhibitors (AI)—letrozole and exemestane. A cell viability assay, a flow cytometer analysis of apoptosis and cell cycle phases, and protein activity of BAX and Bcl-2 were performed on two human breast cancer cell lines—MCF-7 and MCF-7/DOX. In MCF-7 cells, the lower concentration of exemestane and higher of letrozole, in combination with metalloestrogens, results in a decrease in the effectiveness of drugs. Additionally, in the MCF-7/DOX cell line, we observed that the combination of metalloestrogens and AI leads to a decrease in the drug’s effectiveness due to an increase in the viability of breast cancer cells (both concentrations of letrozole and higher concentration of exemestane). In both cell lines, the reduction in the effectiveness of AI, in combination with metalloestrogens, is not related to the influence on the cell cycle. Our results confirm that exposure to metalloestrogens may negatively affect the effectiveness of hormone therapy with AI. Further studies are needed to fully explain the mechanism of these interactions.

## 1. Introduction

In 2020, breast cancer (BC) was diagnosed in 2.3 million women worldwide, and according to the latest prognosis of the American Cancer Society, in 2022 breast cancer will continue to be the most common cancer among women [1,2]. Breast cancer is not a homogeneous disease depending on the expression of key receptors. Several subtypes are distinguished, which differ in the treatment method and prognosis [3]. One of the more common subtypes is steroid receptor-expressing hormone-dependent breast cancer, which is diagnosed in up to 70% of breast cancer patients [4]. In the treatment of hormone-dependent breast cancer, apart from surgery, radiotherapy, and chemotherapy, long-term use of hormone therapy is crucial to eliminate the proliferation-inducing effects of estrogens [3]. In early breast cancer, tamoxifen for 5–10 years is predominantly used in premenopausal women, and tamoxifen, aromatase inhibitors, or their sequence in postmenopausal women. Aromatase inhibitors or fulvestrant in combination with a cyclin-dependent kinase inhibitor CDK4/6, e.g., palbocyclib or alone—tamoxifen, aromatase inhibitors, or high doses (500 mg i.m.) of fulvestrant are used for hormonal treatment in patients with advanced breast cancer [5,6,7]. In recent years, treatment with aromatase inhibitors has become more and more popular due to their higher efficacy and a good tolerance profile compared to the current gold standard—tamoxifen. The mechanism of action of aromatase inhibitors is based on the inhibition of the activity of the aromatase enzyme, which is involved in the conversion of androgens to estrogens. The effect of their action is the elimination of estrogens stimulating the proliferation of cancer cells [5,8].

Xenoestrogens, also called endocrine-disrupting compounds (EDCs), are exogenous substances that disrupt the functioning of the endocrine system and exhibit estrogen-like effects, interacting with estrogen receptors (they act as their antagonists or agonists), interfering with the synthesis and metabolism of endogenous estrogens, and influencing the synthesis of estrogen receptors [9,10,11]. There are several major classes of xenoestrogens—phytoestrogens (e.g., genistein), mycoestrogens (e.g., zearalenone), pesticides, pharmaceuticals, and industrial chemicals (e.g., dichlorodiphenyltrichloroethane—DDT, diethylstilbestrol—DES), synthetic compounds and detergents (e.g., bisphenol A—BPA), and metalloestrogens (e.g., cadmium, chromium, and aluminum) [12]. The Endocrine Society highlights the link between different pathologies, including hormone-dependent cancers in women, and different EDCs, including BPA and dioxins [13,14,15,16,17]. Much less is known about the influence of xenoestrogens on the effectiveness of breast cancer treatment. Several studies have shown that genistein can reverse the therapeutic effect of tamoxifen and its active metabolite—4-hydroxytamoxifen [18,19,20,21,22,23,24]. Genistein and zearalenone reversed the inhibitory effect of palbociclib + letrozole combination on cancer cell proliferation, and BPA antagonizes the cytotoxicity of chemotherapeutic drugs (doxorubicin, cisplatin, and vinblastine) in both ER-positive and ER-negative breast cancer cells [25,26].

Despite the common use of metalloestrogens in everyday life, e.g., aluminum and chromium (III), there is no research on how these metalloestrogens affect the effectiveness of breast cancer hormone therapy [11]. Although the human population may be exposed to aluminum from a range of sources, including diet, antacids, and vaccine adjuvants, frequent application of antiperspirants with aluminum salts to the under-arm region adds a relatively high additional exposure directly to the local area of the human breast. Coincidentally, this is also the region of the human breast where there is a disproportionately high incidence of both breast cysts and breast cancer. Aluminum-based antiperspirants (mainly with aluminum chloride or aluminum chlorohydrate) are applied regularly, often to skin irritated by shaving, which further increases exposure to this metalloestrogen [27]. In addition, Darbre and her colleagues have shown that long-term exposure to aluminum (10^−4^ M aluminum chloride or aluminum chlorohydrate) can increase migratory and invasive properties of MCF-7 human breast cancer cells [28]. Of the two environmentally available forms of chromium, hexavalent and trivalent, the hexavalent form has been classified by IARC (the International Agency for Research on Cancer) as a human carcinogen and mutagen. There are conflicting results in the literature concerning the cytotoxicity and genotoxicity of chromium (III). Chromium (III) salts and chromium (III) compounds have been shown to induce DNA damage, sister chromatid exchange, centromere positive and negative micronuclei, oxidative damage, and Cr–DNA adducts [29]. Moreover, some in vitro studies show that chromium chloride has estrogenic activity. Estrogenicity is defined as the property of producing biological responses qualitatively similar to those produced by the endogenous hormone, 17β-estradiol [16]. The effect of chromium as an endocrine disruptor is becoming increasingly important due to the popularity of trivalent chromium taken in the form of dietary supplements or over-the-counter medications. They are used to regulate glucose levels or to reduce weight; although the available data are inconclusive [30].

The purpose of this study was to analyze the interaction of selected metalloestrogens, aluminum and chromium (III), and drugs used in the treatment of hormone-dependent breast cancer—aromatase inhibitors (AI): non-steroidal AI—letrozole and steroidal AI—exemestane. Our research was conducted on the two estrogen-dependent breast adenocarcinoma cell lines: MCF-7 and MCF-7/DOX (doxorubicin-resistant cell lines). Due to widespread exposure to metalloestrogens, as well as a steady increase in the incidence of BC, examining their impact on the effectiveness of therapies used in the treatment of hormone-dependent BC is becoming a clinically important issue.

## 2. Materials and Methods

### 2.1. Materials

DMEM, low-glucose (Dulbecco’s modified eagle’s medium), FBS (fetal bovine serum), penicillin-streptomycin (10×) solution, and PBS (phosphate-buffered saline) were purchased from Biological Industries, Haemek, Israel. Aluminum chloride hydrate, chromium (III) chloride hexahydrate, letrozole, exemestane, testosterone, DMSO (dimethyl sulfoxide), deionized water, and ethanol were obtained from Sigma Aldrich, Burlington, MA, USA. TrypLE^TM^ Express and GlutaMAX^TM^ were from Gibco, Waltham, MA, USA. Annexin V-FITC Apoptosis Kit and FxCycle^TM^ PI/RNase Staining Solution were purchased from Invitrogen, Waltham, MA, USA. Cell Proliferation Kit II (XTT) was from Roche Diagnostics, Mannheim, Germany. The Halt^TM^ Protease Inhibitor Coctail (100×) and Pierce^TM^ BCA Protein Assay Kit were obtained from Thermo Fisher Scientific, Waltham, MA, USA. The lysis buffer for ELISA, Nori Human Apoptosis Regulator BAX ELISA Kit, and Nori Human Bcl-2 ELISA Kit were purchased from Genorise Scientific, Glen Mills, PA, USA. Accutase^TM^ Cell Detachment was obtained from BD Biosciences, San Jose, CA, USA.

### 2.2. Methods

#### 2.2.1. Cell Culture

Estrogen-dependent breast adenoma cell line, MCF-7, was purchased in CLS Cell Lines Service GmbH, Eppelheim, Germany. The MCF-7/DOX (an MCF-7 cell line with P-gp overexpressing; a doxorubicin-resistant cell line) was derived from an MCF-7 cell line by 3-month cultivation in the presence of a low doxorubicin concentration. Cells were cultured in complete DMEM growth medium (DMEM, low-glucose with glucose concentration 5.5 (5) mM), supplemented with fetal bovine serum (FBS)—10% *v*/*v*, 2 mM L-glutamine, anti-biotics streptomycin (10,000 U/mL), penicillin (10 mg/mL), and 10^−9^ M testosterone, at 37 °C in a humidified atmosphere with 5% CO_2_. The cells were subcultured twice a week using TrypLE^TM^ Express.

#### 2.2.2. Drugs and Metalloestrogens Solutions

Letrozole (LET) and exemestane (EXE) were dissolved in DMSO as 100 mM stock solution (LET: 285 mg in 10 mL DMSO and EXE: 296 mg in 10 mL DMSO) and stored at −20 °C. Aluminum chloride hydrate (AL) and chromium (III) chloride hexahydrate (CR) were dissolved in deionized water as 1 mM stock solution and stored at −20 °C. The working solutions were freshly prepared before each experiment by dilution of stock solution in a culture medium. To prepare the highest concentration of EXE (200 µM) and LET (100 µM), we used 10 µL of stock solution of EXE and 5 µL of stock solution of LET, respectively, so the final DMSO concentration was less than 0.2% in EXE and less than 0.1% in LET at the highest aromatase inhibitor concentration tested.

#### 2.2.3. Cell Viability Assay

The cytotoxic effect of letrozole, exemestane, chromium (III) salt, aluminum (III) salt, and their combinations on MCF-7 and MCF-7/DOX cells was determined using the XTT assay. The XTT assay is based on the cleavage of the yellow tetrazolinum salt XTT (sodium 3′-[1-(phenylaminocarbonyl)-3,4-tetrazolinum]-bis (4-methoxy-6-nitro) benzene sulfonic acid hydrate) to form an orange formazan dye by metabolic active cells. Briefly, 1 × 10^4^ cells were seeded in a 96-well plate and treated in triplicates with various concentrations of letrozole (10 nM–100 µM), exemestane (12.5–200 µM), and chromium (III)/aluminum (III) salt (5–250 µM) for 72 h. To determine the cytotoxicity of the drug–metalloestrogen combination, we treated cells with the drugs (in two different doses) and metalloestrogen (EXE1 = 100 µM; EXE2 = 200 µM; LET1 = 10 µM; LET2 = 100 µM) + 100 µM CR/AL for 72 h. Untreated control cells were also included. Cells supplemented with 0.2% DMSO were considered as a control. After incubation, the XTT assay was performed according to the manufacturer’s instructions. Absorbance was determined at λ = 450 nm, with a reference wavelength at 650 nm using Synergy HTX Multi-Mode Microplate Reader, BioTek, Winooski, VA, USA. The untreated control served as the 100% reference.

#### 2.2.4. Apoptosis and Necrosis Assay

Apoptosis and necrosis were detected with flow cytometry. Cells were stained with an Annexin V-FITC Apoptosis Kit, which contains annexin V conjugated to fluorescein (FITC, annexin V) and propidium iodide (PI). The staining allows for the distinguishing of living cells, early and late apoptotic cells, and necrotic cells. Briefly, 1 × 10^6^ cells were seeded in a 6-well plate and treated with letrozole (LET 1 = 10 µM; LET 2 = 100 µM), exemestane (EXE1 = 100 µM; EXE2 = 200 µM), Cr(III)/Al(III) salt (100 µM) and their combination for 72 h. The untreated control was also prepared and cells supplemented with 0.2% DMSO were considered as a control. Following incubation, the cells were detached with Accutase^TM^ Cell Detachment and washed in cold PBS. The cells were resuspended in 100 µL 1 × annexin-binding buffer and stained with 5 µL of FITC annexin V and 1 µL of PI. After the incubation period (15 min in the dark at room temperature) 400 µL 1 × annexin-binding buffer was added and samples were immediately analyzed in the flow cytometer (CyFlow^®^ SPACE flow cytometer, Sysmex, Kobe, Japan).

#### 2.2.5. Cell Cycle Analysis

To study the anti-proliferative effects induced by the AIs, metalloestrogens, and their combinations, cell cycle analysis was performed with flow cytometry. Cells were stained with FxCycle^TM^ PI/RNase Staining Solution, which allows for the measurement of DNA content and cell distribution among three major phases of the cell cycle: G0/G1, S, and G2/M. The number of cells, the concentrations used, and the incubation time was the same as for the apoptosis and necrosis assay (Section 2.2.4). After incubation, the cells were detached with TrypLE^TM^ Express solution and washed with cold PBS. The cells were then fixed with ice-cold 70% ethanol and kept on ice for 1 h. After two washing steps with cold PBS, the cells were resuspended in 500 µL of PI solution and incubated for 30 min, protected from light. Following incubation, samples were analyzed in the flow cytometer (CyFlow^®^ SPACE flow cytometer, Sysmex, Kobe, Japan).

#### 2.2.6. Flow Cytometric Analysis 

All cytometric analyzes were performed on CyFlow^®^ SPACE flow cytometer (Sysmex, Japan). The laser excitation 488 nm (50 mW) and the filter 536/40 (BP) were used for fluorescence measurement of FITC. Propidium iodide fluorescence was measured using laser excitation 488 nm (50 mW) and the filter 675/20 (BP). The MultiCycle^TM^ DNA analysis model was used for cell cycle analysis. All results were analyzed using FCS Express 7 Cytometry software (De Novo Software, Pasadena, CA, USA).

#### 2.2.7. Preparation of Cell Lysates

Briefly, 1 × 10^6^ cells per T-25 flask were seeded and treated with letrozole (LET 1 = 10 µM; LET 2 = 100 µM), exemestane (EXE1 = 100 µM; EXE2 = 200 µM), CR/AL (100 µM), and their combination for 48 h. The untreated cell control was also prepared and cells supplemented with 0.2% DMSO were considered as a control. Following incubation, the cells were detached with TrypLE^TM^ Express solution, washed with cold PBS, and transferred to a microfuge tube. The cells were centrifuged to pellet the cells and then any remaining buffer was removed. A 0.5 mL lysis buffer, supplemented with a protease inhibitor cocktail, was added to the cell pellet, vortexed, and incubated for 30 min on ice. Then, samples were centrifuged at 10,000× *g* for 10 min. The supernatants were transferred to a clean tube and stored at −80 °C for further analysis.

#### 2.2.8. Determination of Total Protein Concentration in Cell Lysates

Total protein concentration was determined using the Pierce^TM^ BCA Protein Assay Kit (Thermo Fisher Scientific, Waltham, MA, USA). The assay was performed according to the manufacturer’s instructions. Absorbance was determined at λ = 562 nm using a Synergy HTX Multi-Mode Microplate Reader, BioTek, USA.

#### 2.2.9. ELISA Assays for Bcl-2 and BAX Proteins Detection 

ELISA was used to detect and quantify from cell lysates (details in Section 2.2.7) Bcl-2 and BAX apoptotic proteins involved in the cell death pathway. A Nori Human Apoptosis Regulator BAX ELISA Kit and a Nori Human Bcl-2 ELISA Kit (Genorise Scientific, Glen Mills, PA, USA) were performed, according to the manufacturer’s instructions. Each sample was assayed in duplicate and expressed relative to the total protein concentration in the same sample. Absorbance was determined at λ = 570 nm using a Synergy HTX Multi-Mode Microplate Reader, BioTek, USA. The four-parameter logistic fitted standard curves for calculating the concentration of Bcl-2 and BAX protein were generated from the Arigo Biolaboratories website (https://www.arigobio.com/elisa-analysis, accessed on 15 September 2022). Bcl-2 and BAX concentrations were calculated per 100 µL of total protein in the sample, and the Bcl-2/BAX ratio was then calculated.

#### 2.2.10. Statistical Analysis

Results were analyzed using GraphPad Prism 9 (GraphPad Software, Boston, MA, USA) using one-way ANOVA, followed post hoc by Tukey’s (or Dunnett’s tests in case of cell viability assay for alone compounds) multiple comparisons tests. The results were expressed as the mean and standard deviation of the mean (SD). Each experiment was repeated three times. Significant differences among means were estimated at *p* < 0.05.

## 3. Results

### 3.1. Effect of Metalloestrogens, Aromatase Inhibitors, and Their Combination on Cell Viability

#### 3.1.1. Metalloestrogens and Aromatase Inhibitors Alone

Results obtained for individual compounds—exemestane (12.5–200 µM), letrozole (10 nM–100 µM), chromium (III), and aluminum (5–250 µM), are in the Supplementary Materials (Figures S1 and S2). Exemestane and letrozole were more cytotoxic to MCF-7 cells than to MCF-7/DOX cells, with exemestane being more potent in both cell lines. Aluminum and chromium (III) had no cytotoxic effect; they stimulated cell proliferation and there were more MCF-7 cells than MCF-7/DOX cells.

#### 3.1.2. Metalloestrogens and Aromatase Inhibitors in Combination

**Figure 1:**
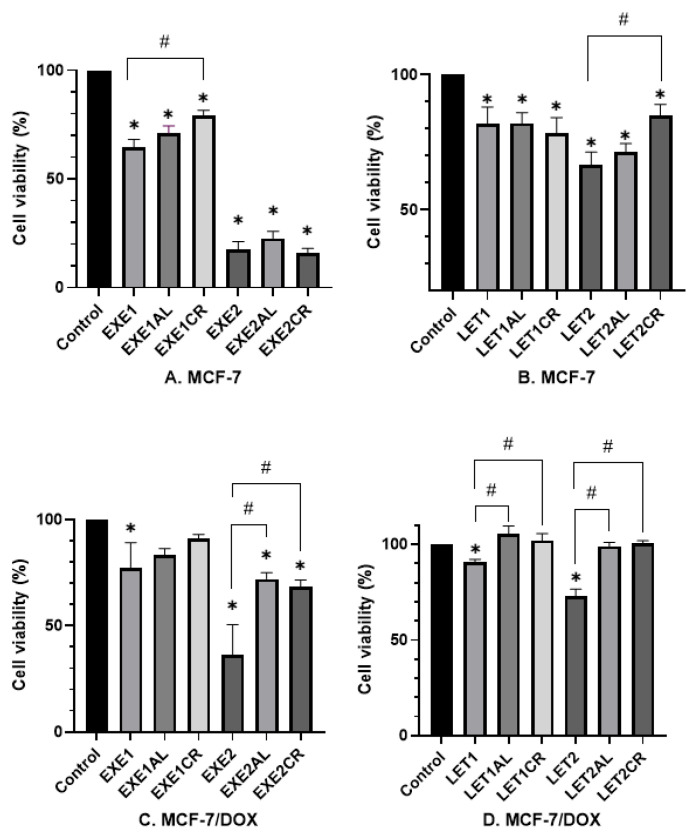
Effect of metalloestrogens and aromatase inhibitors in combination on cell viability. The viability of MCF-7 cells treated with exemestane (**A**) and letrozole (**B**), alone or in combination with aluminum or chromium (III). The viability of MCF-7/DOX cells treated with exemestane (**C**) and letrozole (**D**), alone or in combination with aluminum or chromium (III). EXE1 = 100 µM; EXE2 = 200 µM; LET1 = 10 µM; LET2 =100 µM; Al/Cr(III) = 100 µM. The results are presented as mean ± SD, *n* = 3; *p* < 0.05; * statistically significant difference from the control; # statistically significant difference from the aromatase inhibitor.

The results are shown in Figure 1.

- Effects on MCF-7 cells.

The combination of exemestane (100 µM) with both aluminum and chromium (III) increased the viability of MCF-7 cells (EXE1—65%; EXE1AL—71%; and EXE1CR—79%, respectively). The result for chromium (III) is statistically significant (*p* = 0.0004). The combination of exemestane at 200 µM with metalloestrogens had no significant effect (EXE2 18% vs. EXE2AL 23%, *p* = 0.3888; vs. EXE2CR 16%, *p* = 0.997) on MCF-7 cells viability. Letrozole (10 µM) in combination with aluminum or chromium (III) had no statistically significant effect on MCF-7 cell viability (LET1 82% vs. LET1AL 82%, *p* > 0.9999; vs. LET1CR 78%, *p* = 0.9645). However, a combination letrozole at a concentration of 100 µM with chromium (III) and aluminum increased cell viability (LET2—67%; LET2CR—85%; and LET2AL—70%, respectively). The difference between LET2 and LET2CR was statistically significant (*p* = 0.0028).

- Effects on MCF-7/DOX cells.

Aromatase inhibitors were less cytotoxic to MCF-7/DOX cells than MCF-7 cells. The combination of EXE1 with aluminum or chromium (III) increased cell viability, but the results were not statistically significant (EXE1 77% vs. EXE1AL 83%, *p* = 0.9331; vs. EXE1CR 91%, *p* = 0.3006). The combination of EXE2 with metalloestrogens was statistically significant and increased the viability of MCF-7/DOX cells—EXE2 37% vs. EXE2AL 72%, *p* = 0.0006; vs. EXE2CR 68%, *p* = 0.0017. The combination of letrozole (in both concentrations) with metalloestrogens significantly increased the viability of MCF-7/DOX cells: LET1 91% vs. LET1AL 106%, *p* = 0.0001; vs. LET1CR 102%, *p* = 0.0022; LET2 73% vs. LET2AL 99%, *p* < 0.0001; vs. LET2CR 100%, *p* < 0.0001.

### 3.2. The Effect of Metalloestrogens on Proapoptotic and Necrotic Effects of Aromatase Inhibitors

**Figure 2:**
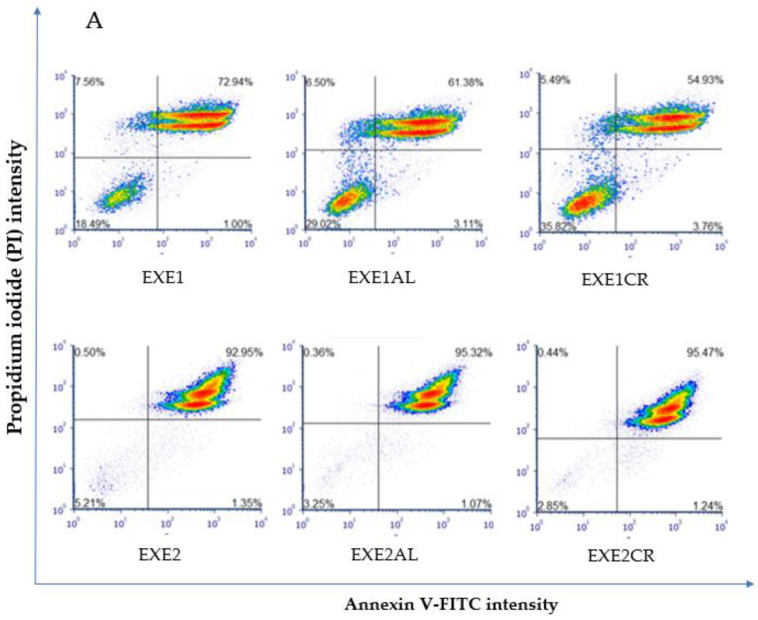

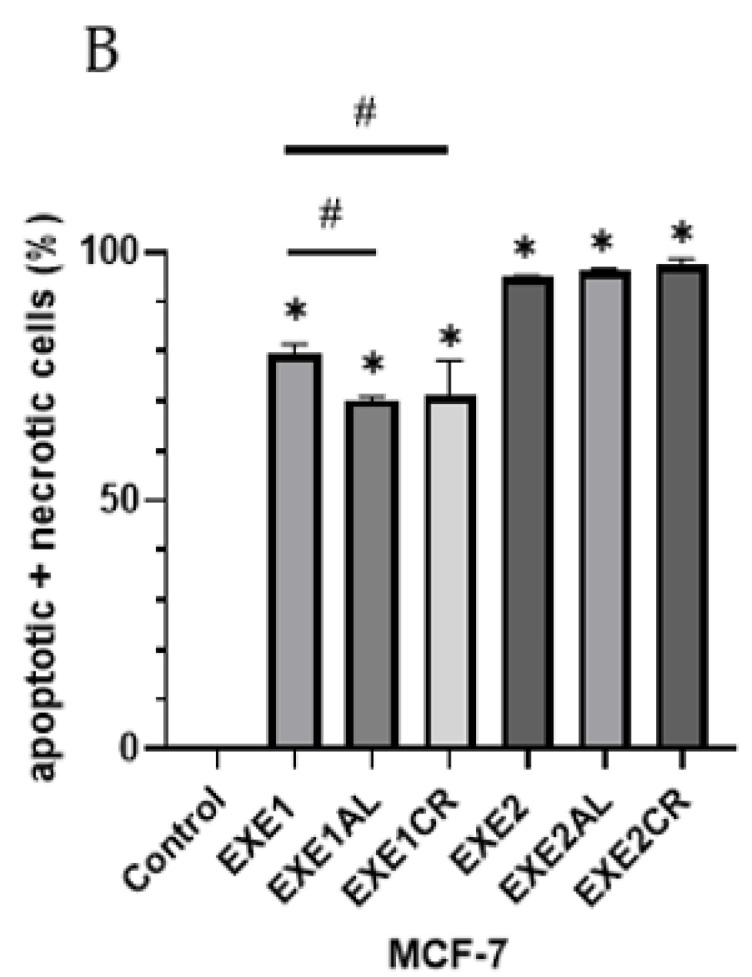
Effect of exemestane alone and in combination with metalloestrogens on MCF-7 cell death. (**A**) Representative cytograms of flow cytometric are shown. EXE1 = 100 µM; EXE2 = 200 µM; Al./Cr(III) = 100 µM. (**B**) Percentage of both apoptotic (early and late apoptotic) and necrotic cells. The results are presented as mean ± SD, *n* = 3; *p* < 0.05; * statistically significant difference from control; # statistically significant difference from aromatase inhibitor alone.

**Figure 3:**
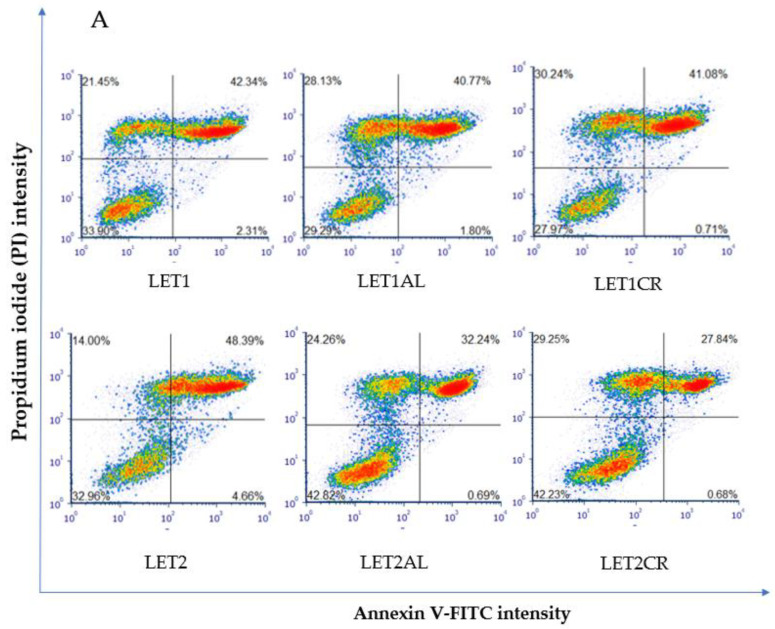

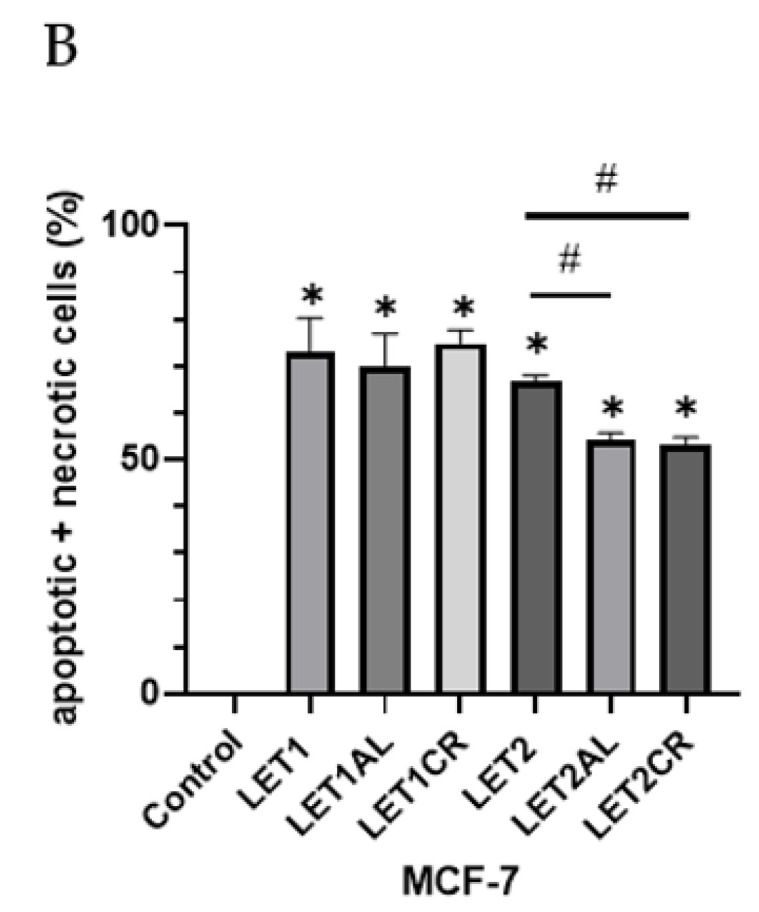
Effect of letrozole alone and in combination with metalloestrogens on MCF-7 cell death. (**A**) Representative cytograms of flow cytometric are shown. LET1 = 10 µM; LET2 = 100 µM; Al./Cr(III) = 100 µM. (**B**) Percentage of both apoptotic (early and late apoptotic) and necrotic cells. The results are presented as mean ± SD, *n* = 3; *p* < 0.05; * statistically significant difference from control; # statistically significant difference from aromatase inhibitor alone.

**Figure 4:**
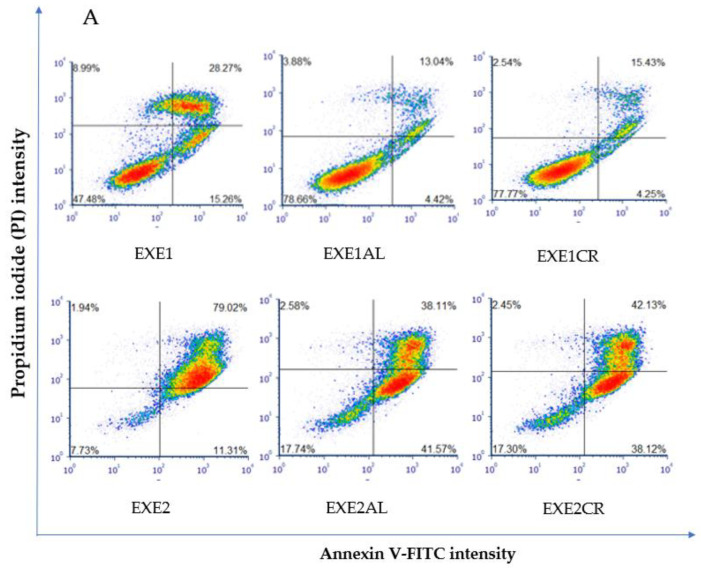

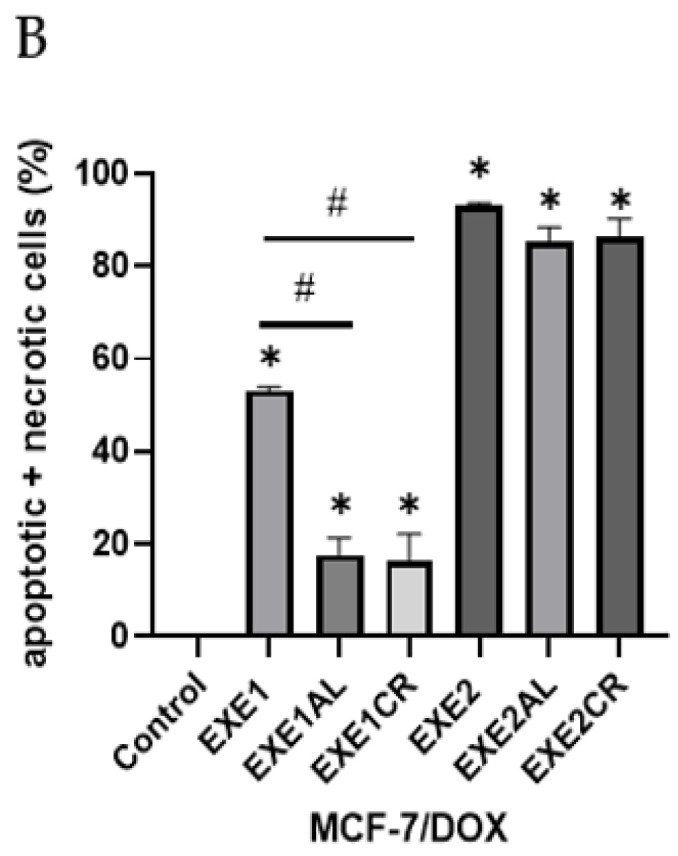
Effect of exemestane alone and in combination with metalloestrogens on MCF-7/DOX cell death. (**A**) Representative cytograms of flow cytometric are shown. EXE1 = 100 µM; EXE2 = 200 µM; Al./Cr(III) = 100 µM. (**B**) Percentage of both apoptotic (early and late apoptotic) and necrotic cells. The results are presented as mean ± SD, *n* = 3; *p* < 0.05; * statistically significant difference from control; # statistically significant difference from aromatase inhibitor alone.

**Figure 5:**
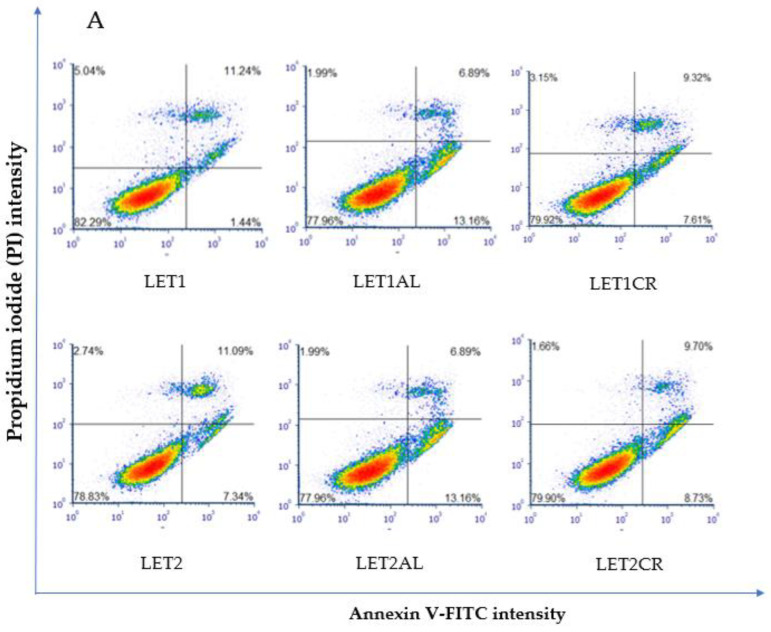

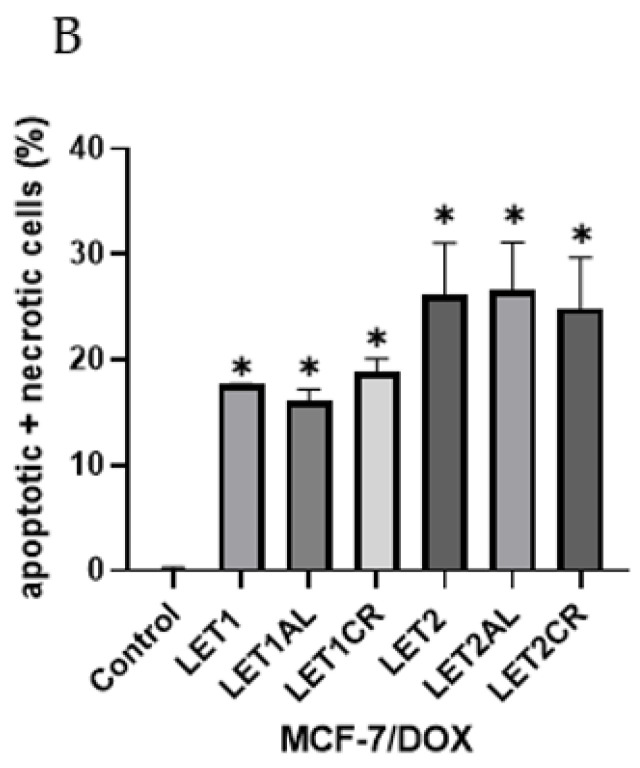
Effect of letrozole alone and in combination with metalloestrogens on MCF-7/DOX cell death. (**A**) Representative cytograms of flow cytometric are shown. LET1 = 10 µM; LET2 = 100 µM; Al./Cr(III) = 100 µM. (**B**) Percentage of both apoptotic (early and late apoptotic) and necrotic cells. The results are presented as mean ± SD, *n* = 3; *p* < 0.05; * statistically significant difference from control.

Proapoptotic and necrotic effects of aromatase inhibitors and their combination with metalloestrogens were studied after 72 h of incubation in MCF-7 and MCF-7/DOX cells using staining with Annexin V-FITC and PI. Results are presented as a percentage of early apoptotic (Annexin V-FITC+, PI−), late apoptotic (Annexin V-FITC+, PI+), and necrotic (Annexin V-FITC−, PI+) cells in Figure 2, Figure 3, Figure 4 and Figure 5. The gating strategy for flow cytometry analysis of apoptosis and necrosis assays is in the Supplementary Materials (Figure S3). A representative cytogram for the controls is in the Supplementary Materials (Figures S4 and S5).

#### 3.2.1. Effects on MCF-7 Cells

The combination of 100 µM exemestane with metalloestrogens significantly reduced the percentage of cells undergoing apoptosis and necrosis (EXE1 vs. EXE1AL, *p* = 0.0152; vs. EXE1CR, *p* = 0.0353). For exemestane at the higher concentration (200 µM), no statistically significant effect of metalloestrogens on the aromatase inhibitor efficacy was observed (EXE2 vs. EXE2AL, *p* = 0.9973; vs. EXE2CR, *p* = 0.9305). The results are shown in Figure 2.

The combination of 10 µM of letrozole with metalloestrogens had no effect on the proapoptotic and necrotic activity of aromatase inhibitor (LET1 vs. LET1AL, *p* = 0.9540; vs. LET1CR, *p* = 0.9982). However, the combination of a higher concentration of letrozole (100 µM) with metalloestrogens significantly reduced the percentage of necrotic and apoptotic cells, thus reducing the effectiveness of the drug (LET2 vs. LET2AL, *p* = 0.0224; vs. LET2CR, *p* = 0.0125). The results are shown in Figure 3.

#### 3.2.2. Effects on MCF-7/DOX Cells

The combination of exemestane at 100 µM with metalloestrogens significantly reduced the number of apoptotic/necrotic MCF-7/DOX cells (EXE1 vs. EXE1AL, *p* = 0.0152; vs. EXE1CR, *p* = 0.0353). No similar relationship is observed when combining a higher concentration of exemestane (200 µM) and metalloestrogens (EXE2 vs. EXE2AL, *p* = 0.1097; vs. EXE2CR, *p* = 0.2246). The results are shown in Figure 4.

Letrozole, at both concentrations, in combination with metalloestrogens, did not have a statistically significant effect on the proapoptotic/necrotic effects of the drug (LET1 vs. LET1AL, *p* = 0.9960; vs. LET1CR, *p* = 0.9992; LET2 vs. LET2AL, *p* > 0.9999; vs. LET2CR, *p* = 0.9989). The results are shown in Figure 5.

### 3.3. The Effect of Aromatase Inhibitors and Their Combination with Metalloestrogens on the Cell Cycle

To determine whether the combination of aromatase inhibitors and metalloestrogens may disturb the cell cycle progression, we further evaluated their effects on the cell cycle. For this purpose, after 72 h of treatment, the cells were stained with PI and the percentage of cells in the G0/G1, S, and G2⁄M phases were determined using flow cytometry.

#### 3.3.1. Exemestane

Exemestane induces cell cycle arrest in the G0/G1 phase and G2/M phase, and reduces the number of cells in the S phase. In the MCF-7 cell line, a combination of exemestane (100 µM) with aluminum results in an increase in the number of cells in the S phase (and a decrease in the G2/M phase). A combination of metalloestrogens (both aluminum and chromium (III) with exemestane in higher concentration (200 µM) results in an increase in the percentage of cells in the G2/M phase (compared to EXE2). In the MCF-7/DOX cell line, a combination of exemestane (200 µM) with aluminum or chromium (III) results in an increase in the number of cells in the S phase. However, in both lines, MCF-7 and MCF-7/DOX, changes in the distribution of cells in individual phases of the cell cycle are non-statistically significant. The results are presented in Table 1.

#### 3.3.2. Letrozole

Letrozole also induces cell cycle arrest in the G0/G1 phase. In both cell lines, MCF-7 and MCF-7/DOX, a combination of letrozole with metalloestrogens results in non-statistically significant changes in the distribution of cells in phases of the cell cycle. In the MCF-7/DOX cell line, the combination of letrozole (10 µM) with aluminum increases the percentage of cells in the G2/M phase, but the result is not statistically significant. The results are presented in Table 2.

### 3.4. The Effect of Aromatase Inhibitors and Their Combination with Metalloestrogens on Bcl-2/BAX Ratio

Bcl-2 and BAX concentrations were calculated per 100 µL of total protein in the sample; then the Bcl-2/BAX ratio was calculated. In the case of the MCF-7/DOX cell line, lower concentrations of BAX protein (pro-apoptotic protein) and similar (to MCF-7 cell line) concentrations of Bcl-2 protein (anti-apoptotic protein) were observed, resulting in higher Bcl-2/BAX ratios. An increase in the Bcl-2/BAX ratio indicates a reduced susceptibility of cells to apoptosis, and thus a reduction in the effectiveness of the aromatase inhibitor.

#### 3.4.1. The Effect of the Combination of Exemestane and Metalloestrogens on the Bcl-2/BAX Ratio

**Figure 6:**
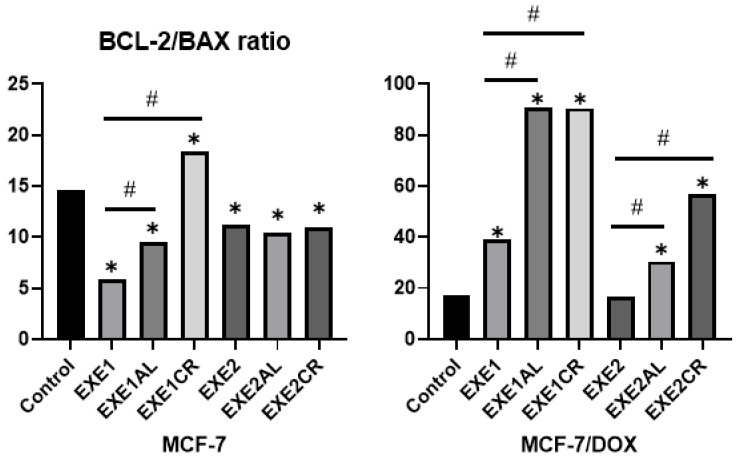
The effect of exemestane and its combination with metalloestrogens in the Bcl-2/BAX ratio. EXE1 = 100 µM; EXE2 = 200 µM; and Al/Cr(III) = 100 µM. The results are presented as mean, *n* = 3; *p* < 0.05; * statistically significant difference from control; # statistically significant difference from aromatase inhibitor alone.

In both lines, the combination of a lower concentration of exemestane (100 µM) with metalloestrogens resulted in a statistically significant increase in the Bcl-2/BAX ratio. In the case of a higher concentration of exemestane (200 µM), a statistically significant increase in the Bcl-2/BAX ratio was observed only in the MCF-7/DOX line. The results are shown in Figure 6 and Table 3.

#### 3.4.2. The Effect of the Combination of Letrozole and Metalloestrogens on the Bcl-2/BAX Ratio

**Figure 7:**
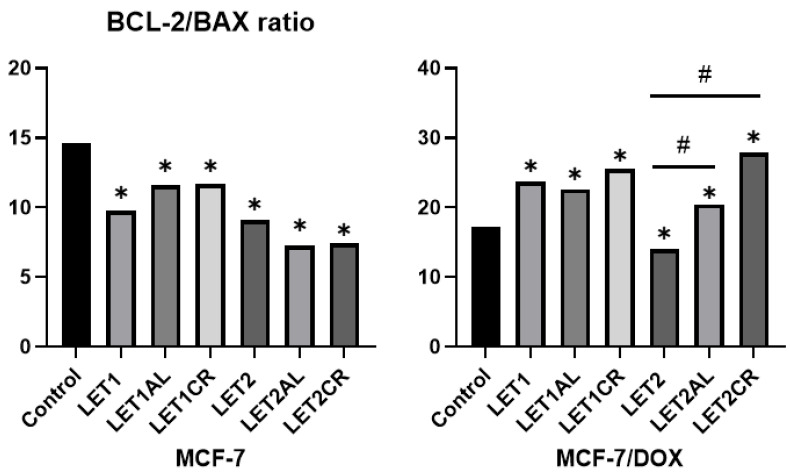
Effect of letrozole and its combination with metalloestrogens in the Bcl-2/BAX ratio. LET1 = 10 µM; LET2 = 100 µM; and Al/Cr(III) = 100 µM. The results are presented as mean, *n* = 3; *p* < 0.05; * statistically significant difference from control; # statistically significant difference from aromatase inhibitor alone.

Incubation of MCF-7 cells with the combination of letrozole (at both concentrations) and metalloestrogens did not result in a statistically significant increase in the Bcl-2/BAX ratio, while in MCF-7/DOX cells, the combination of higher concentrations of letrozole with metalloestrogens increased it. The results are shown in Figure 7 and Table 4.

## 4. Discussion

Hormone-dependent breast cancer is the most commonly diagnosed subtype, especially in postmenopausal women [4]. Most patients with hormone-dependent breast cancer, except for hormone therapy, also receive preoperative or postoperative chemotherapy based on the sequential use of multi-drug regimens based on cytostatics—anthracyclines (e.g., doxorubicin and epirubicin) and taxoids (e.g., docetaxel and paclitaxel). Doxorubicin is currently the most effective and popular chemotherapeutic drug used to treat breast cancer. Unfortunately, resistance to this agent is common, representing a major obstacle to successful treatment [31]. In our study, we performed tests on two cell lines: MCF-7 and MCF-7/DOX, lines resistant to doxorubicin, because patients using hormone therapy often had prior chemotherapy which could lead to the selection of cancer cells resistant to doxorubicin. Therefore, we want to compare the response of both cell lines to the aromatase inhibitors, metalloestrogens, and their combinations. Studies by Devajaran et al. Indicate that MCF-7/DOX cells were exquisitely sensitive to apoptotic stimuli. Thus, treatment with staurosporine (50 nM for 48 h) consistently exerted higher cytotoxicity against MCF-7/DOX cells when compared to the MCF-7 cells under similar conditions, indicating a differentiated response between lines to the same stimuli [32]. We confirmed that the MCF-7 and MCF-7/DOX cell lines reacted differently to the same stimuli; however, in our studies it was the MCF-7/DOX cells that were less susceptible to the cytotoxic effects of drugs (as well as their combination with metalloestrogens) and to apoptosis. It was manifested, among others, by lower concentrations of the BAX protein and an increased Bcl-2/BAX ratio, which indicates a lower susceptibility to apoptosis [33]. At the same time, MCF-7/DOX cells were less susceptible to the stimulating effect of metalloestrogens.

In this study, we attempted to assess whether there is an interaction between metalloestrogens: aluminum, chromium (III), and aromatase inhibitors (exemestane and letrozole) that reduces the effectiveness of the drugs. To the best of our knowledge, similar issues have not been studied so far, so we can only refer to the effects of single substances or their combinations with other xenoestrogens.

The results of this study showed that a combination of metalloestrogens with exemestane leads to a reduction in its cytotoxicity. In the MCF-7 cell line, a statistically significant decrease in the activity of the drug was visible in the combination of chromium (III) with a lower concentration of exemestane (100 µM), while in the MCF-7/DOX cell line with a the combination of both chromium (III) and aluminum with a higher concentration of exemestane (200 µM). Letrozole had a lower cytotoxic effect than exemestane, but in the case of letrozole, we observed a decrease in drug activity after the addition of metalloestrogens. In the MCF-7 cell line, a decrease in letrozole activity was observed when combining a higher concentration with chromium (III); while in the MCF-7/DOX cell line, a decrease in drug activity was observed in all analyzed combinations. We have identified several studies that investigated the effects of xenoestrogens (from the phytoestrogen group) on the effectiveness of aromatase inhibitors. Ju et al. Studied the effect of genistein on therapy with an aromatase inhibitor, letrozole, as an animal model. While letrozole was effective in inhibiting tumor growth, adding genistein to the mice’s diet reversed this effect [19]. Van Duursen et al. Studied the effect of genistein and 8-prenylnarigenin alone, as well as four multicomponent dietary supplements on the effectiveness of letrozole. Genistein and 8-prenylnarigenin, as well as all tested supplements containing various combinations of phytoestrogens, have been shown to activate an estrogen-dependent increase in MCF-7 cell proliferation that was not inhibited by letrozole [34]. Warth et al. Studied the influence of dietary xenoestrogens (zearalenone and genistein) on the effectiveness of treatment with letrozole and palbociclib. An in vitro study on breast cancer cell lines showed that the combination of letrozole and palbociclib effectively inhibited tumor cell proliferation, but the addition of both genistein and zearalenone counteracted this effect [25]. Different results, i.e., lack of interaction between formestane, a second-generation steroid aromatase inhibitor (currently no longer used in the treatment of breast cancer), and phytoestrogens derived from the root bug extract, were shown in the animal model by Nißlein et al. [35].

Apoptosis is the programmed cell death that occurs in response to various environmental stimuli and can be induced through intrinsic and extrinsic pathways. The intrinsic pathway is regulated by the Bcl-2 family of proteins—a balance between pro-apoptotic proteins, such as BAX, and anti-apoptotic proteins, e.g., Bcl-2 is a key apoptosis regulator in numerous types of cells [36,37]. Previous studies have shown that some xenoestrogens, such as estrogens, can reduce the rate of apoptosis, e.g., bisphenol A (BPA) via increasing the Bcl-2/BAX ratio and di(2-ethylhexyl) phthalate (DEHP) via activating the Akt and NF-κB pathways [14,38,39]. Most drugs used in anticancer therapy, including aromatase inhibitors, lead to the elimination of cancer cells through apoptosis and cell cycle dysregulation [40,41]. Exemestane (a steroidal aromatase inhibitor) decreased MCF-7 cell proliferation, induced cell cycle arrest at G0/G1 and G2/M, as well as apoptosis through the mitochondrial pathway and cytoprotective autophagy [42]. Letrozole (a non-steroidal aromatase inhibitor) also inhibited the growth of breast cancer cells by induced cell cycle arrest at the G0/G1 phase (but without G2/M arrest) and apoptosis by the intrinsic pathway, which was associated, among others, with the decreased Bcl-2 protein expression and the increased BAX protein expression. Non-steroidal aromatase inhibitors (except letrozole and anastrozole), unlike exemestane, did not induce autophagy [41,43].

Our research showed that exemestane induces apoptosis in a dose-dependent manner and this effect is more potent in MCF-7 cells than in MCF-7/DOX cells. In MCF-7 cells, this is associated with a significant decrease in the Bcl-2/BAX ratio. Any agent that decreased the Bcl-2/BAX ratio may promote apoptosis [44]. In MCF-7 cells, the addition of both metalloestrogens, aluminum or chromium (III), to exemestane (100 µM) reduces the percentage of cells undergoing apoptosis. According to our research, responsible for this effect is an increase in the Bcl-2/BAX ratio, mainly related to a decrease in the concentration of the pro-apoptotic protein BAX. Similar relationships are not observed at high concentrations of exemestane (200 µM), whose activity is high regardless of the combination with metalloestrogens, which also had no effect on lowering the Bcl-2/BAX ratio. We obtained similar results in the MCF-7/DOX cell line, where a combination of a lower concentration of exemestane with metalloestrogens resulted in a decrease in the number of cells undergoing apoptosis, followed by an increased Bcl-2/BAX ratio. In the case of high concentrations of exemestane, we did not observe such a relationship. The effect of letrozole, a non-steroidal aromatase inhibitor, also leads to apoptosis of hormone-dependent breast cancer cells and, as in the case of exemestane, this effect is stronger in MCF-7 cells than in MCF-7/DOX, which may be explained by lower concentrations of the pro-apoptotic protein BAX in the MCF-7/DOX cell line (a higher Bcl-2/BAX ratio). Interestingly, we observed that the combination of letrozole and metalloestrogens resulted in a lower negative effect on apoptosis than in exemestane, and a decrease in the number of apoptotic cells was significant only in the MCF-7 line when letrozole (100 µM) was combined with aluminum or chromium (III).

To study the anti-proliferative effects induced by Ais and their combination with metalloestrogens, cell cycle progression was evaluated by flow cytometry. Estrogens induce cell proliferation by stimulating progression through the G0/G1 phase of the cell cycle. We confirmed that both Ais (steroidal and non-steroidal), by blocking the effects of estrogens action, arrest the cell cycle in phase G0/G1 (exemestane and in G2/M), which is in line with what has been previously observed by other authors [41,42,43]. However, we did not observe that the simultaneous exposure to metalloestrogens and aromatase inhibitors changed the distribution of cells in the phases of the cell cycle. This means that the reduction in the activity of aromatase inhibitors in combination with metalloestrogens is not related to the influence on the cell cycle.

## 5. Conclusions

The widespread exposure to xenoestrogens and the constantly increasing number of cases of BC lead to more and more detailed studies of the influence of EDC, not only on the carcinogenesis process, but also on the effectiveness of drugs used in the treatment of breast cancer. In the present study, we aimed to evaluate whether exposure to metalloestrogens commonly present in everyday human life, aluminum and chromium (III), may reduce the effectiveness of aromatase inhibitors used in hormone therapy of breast cancer.

We have shown that the MCF-7 and MCF-7/DOX cell lines reacted differently to the same stimuli, and the MCF-7/DOX cells were less susceptible to the cytotoxic effects of the drugs (as well as their combination with metalloestrogens), and to apoptosis. In MCF-7 cells, the lower concentration of exemestane and higher of letrozole, in combination with metalloestrogens, results in a decrease in the effectiveness of drugs (increases cell viability and reduces apoptosis). Additionally, in the MCF-7/DOX cell line, we observed that the combination of metalloestrogens and aromatase inhibitors led to a decrease in the drug’s effectiveness due to an increase in the viability of breast cancer cells (both concentrations of letrozole and higher concentrations of exemestane). However, in the case of the MCF-7/DOX cell line, the regulation of apoptosis was less likely to be responsible for this effect than in the case of MCF-7 cells. In both cell lines, the reduction in the effectiveness of aromatase inhibitors, in combination with metalloestrogens, is not related to the influence on the cell cycle.

Our results indicate that exposure to metalloestrogens may negatively affect the effectiveness of hormone therapy with aromatase inhibitors. They also show that this is a complex issue. Therefore, further research is needed to fully explain these interactions and be able to effectively counteract them in the treatment of patients with hormone-dependent breast cancer.

## Figures and Tables

**Table 1:** Effect of exemestane and its combination with metalloestrogens on cell cycle in MCF-7 and MCF-7/DOX cell lines. EXE1 = 100 µM; EXE2 = 200 µM; and Al/Cr(III) = 100 µM. The results are presented as mean, *n* = 3.

Sample	%G1 (G0/G1)	%S	%G2 (G2/M)
MCF-7			
Control	73.19	24.03	2.78
EXE1	79.51	0.23	20.26
EXE1AL	81.72	7.95	10.33
EXE1CR	84.78	1.65	13.57
EXE2	82.96	6.09	10.95
EXE2AL	76.18	5.70	18.12
EXE2CR	80.43	5.01	14.56
MCF-7/DOX			
Control	63.73	29.22	7.05
EXE1	75.86	0.07	24.07
EXE1AL	76.81	0.00	23.20
EXE1CR	76.14	0.00	23.86
EXE2	71.61	0.62	27.77
EXE2AL	71.70	5.94	22.36
EXE2CR	72.07	3.64	24.29

**Table 2:** The effect of letrozole and its combination with metalloestrogens on the cell cycle in MCF-7 and MCF-7/DOX cell lines. LET 1 = 10 µM; LET 2 = 100 µM; and Al/Cr(III) = 100 µM. The results are presented as mean, *n* = 3.

Sample	%G1 (G0/G1)	%S	%G2 (G2/M)
MCF-7			
Control	73.19	24.03	2.78
LET1	83.00	12.70	4.30
LET1AL	85.48	13.14	1.38
LET1CR	82.03	13.54	4.43
LET2	81.68	14.02	4.30
LET2AL	82.09	17.46	0.45
LET2CR	86.90	13.10	0.00
MCF-7/DOX			
Control	63.73	29.22	7.05
LET1	77.05	21.54	1.41
LET1AL	74.72	18.39	6.88
LET1CR	78.62	20.30	1.08
LET2	73.74	19.79	6.48
LET2AL	77.73	13.93	8.35
LET2CR	80.23	10.47	9.30

**Table 3:** Bcl-2/BAX ratio values in MCF-7 and MCF-7/DOX cell lines after exposure to exemestane alone or in combination with metalloestrogens. The results are presented as mean, *n* = 3; *p* < 0.05.

Sample	Bcl-2/BAX Ratio	*p*
MCF-7		
Control	14.59	
EXE1	5.88	
EXE1AL	9.47	0.0084 * ^1^
EXE1CR	18.44	<0.0001 * ^1^
EXE2	11.22	
EXE2AL	10.46	0.9609 ^2^
EXE2CR	10.99	>0.9999 ^2^
MCF-7/DOX		
Control	17.28	
EXE1	39.15	
EXE1AL	90.72	<0.0001 * ^1^
EXE1CR	90.42	<0.0001 * ^1^
EXE2	16.70	
EXE2AL	30.48	<0.0001 * ^2^
EXE2CR	56.90	<0.0001 * ^2^

^1^ vs. EXE1; ^2^ vs. EXE2; * statistically significant result.

**Table 4:** Bcl-2/BAX ratio values in MCF-7 and MCF-7/DOX cell lines after exposure to letrozole alone or in combination with metalloestrogens. The results are presented as mean, n = 3; *p* < 0.05.

Sample	Bcl-2/BAX Ratio	*p*
MCF-7		
Control	14.59	
LET1	9.76	
LET1AL	11.58	0.3400 ^1^
LET1CR	11.65	0.3020 ^1^
LET2	9.09	
LET2AL	7.27	0.3400 ^2^
LET2CR	7.44	0.4440 ^2^
MCF-7/DOX		
Control	17.28	
LET1	23.71	
LET1AL	22.66	0.8474 ^1^
LET1CR	25.59	0.3073 ^1^
LET2	13.97	
LET2AL	20.44	<0.0001 * ^2^
LET2CR	27.87	<0.0001 * ^2^

^1^ vs. LET1; ^2^ vs. LET2; * statistically significant result.

## Data Availability

All data are available in the article and Supplementary Materials. We will willingly share our knowledge, protocol, and expertise when asked.

## Supplementary Materials

The following supporting information can be downloaded at https://www.mdpi.com/article/10.3390/cancers15020457/s1, Figure S1: The effect of individual metalloestrogens and aromatase inhibitors on MCF-7 cell viability. Figure S2: The effect of individual metalloestrogens and aromatase inhibitors on MCF-7/DOX cell viability. Figure S3: Gating strategy for flow cytometry analysis of apoptosis and necrosis assay. Figure S4: Representative cytogram for MCF-7 cell control. Figure S5: Representative cytogram for MCF-7/DOX cell control.
