# Supplementary material for: The Effect of Metalloestrogens on the Effectiveness of Aromatase Inhibitors in a Hormone-Dependent Breast Cancer Cell Model
Source: Cancers (Basel). 2023 Jan 11;15(2):457. doi: 10.3390/cancers15020457 (PMC9856755; doi:10.3390/cancers15020457)
Supplement: Supplementary file 1 [file cancers-15-00457-s001.zip › cancers-2092413-supplementary.pdf]

SUPPLEMENTARY MATERIALS for:

Article

Effect of metalloestrogens on the effectiveness of aromatase inhibitors in a hormone-dependent breast cancer cell model.

Kamila Boszkiewicz, Helena Moreira, Ewa Sawicka, Anna Szyjka and Agnieszka Piwowar

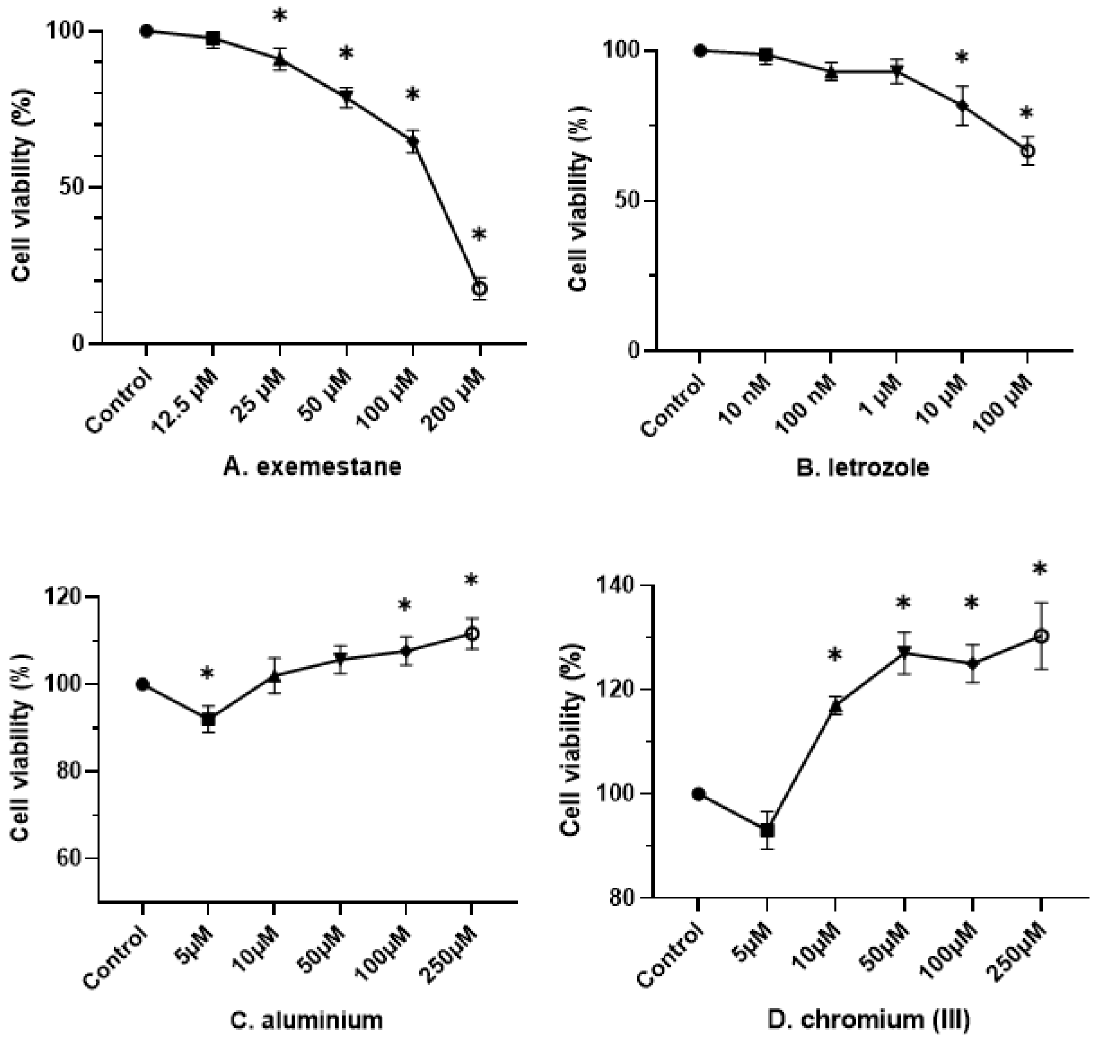

Figure S1. Effect of individual metalloestrogens and aromatase inhibitors on MCF-7 cells viability. The viability of MCF-7 exposed for 72 h for: A. exemestane (12.5-200 µM); B. letrozole (10 nM-100 µM); C. aluminium (5-250 µM) and D. chromium (III) (5-250 µM). The results are presented as mean ± SD, n = 3; $p<0.05$; * statistically significant difference from control.

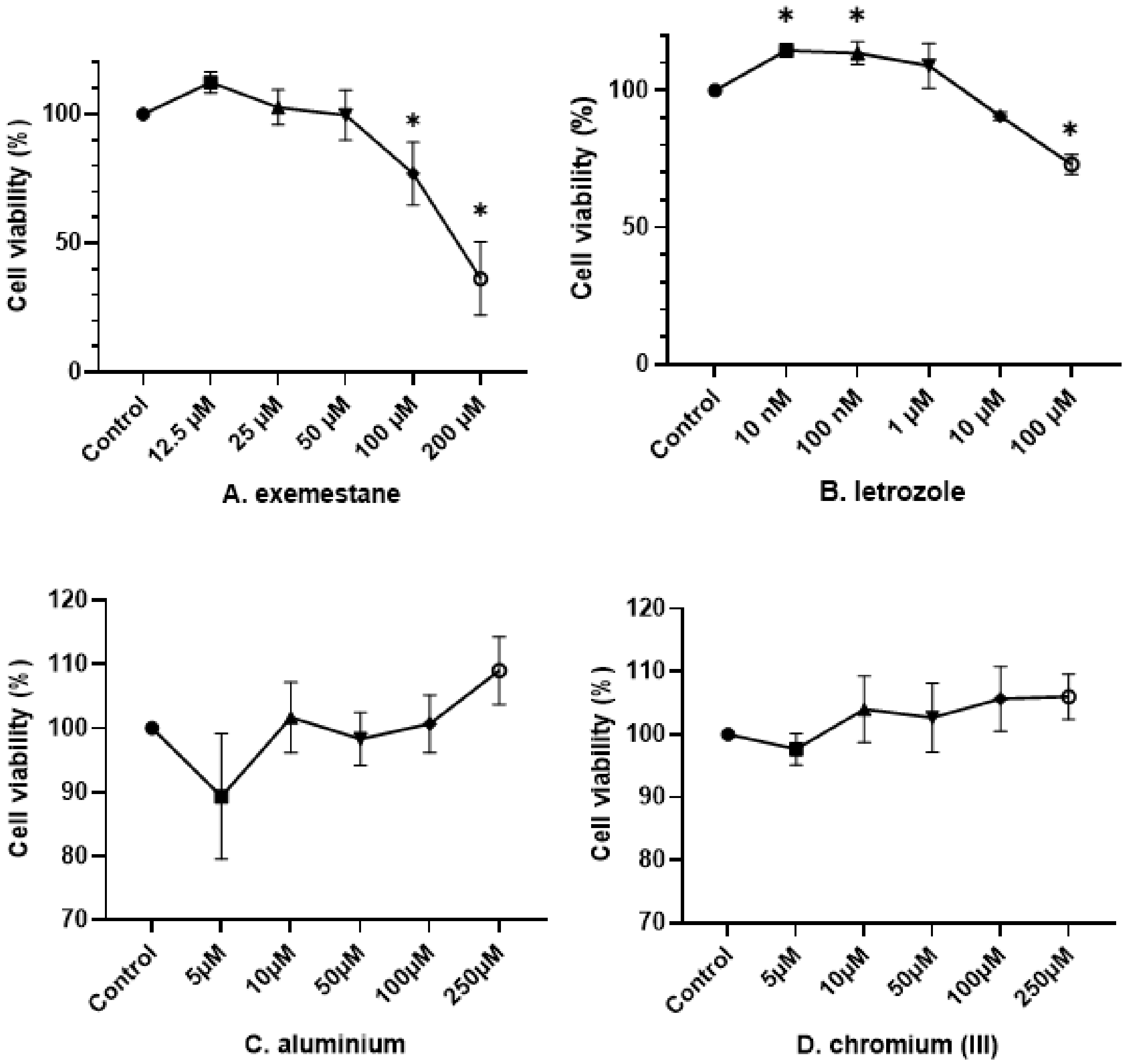

Figure S2. Effect of individual metalloestrogens and aromatase inhibitors on MCF-7/DOX cells viability. The viability of MCF-7/DOX exposed for 72 h for: A. exemestane (12.5-200 µM); B. letrozole (10 nM-100 µM); C. aluminium (5-250 µM) and D. chromium (III) (5-250 µM). The results are presented as mean ± SD, n = 3; $p<0.05$; * statistically significant difference from control.

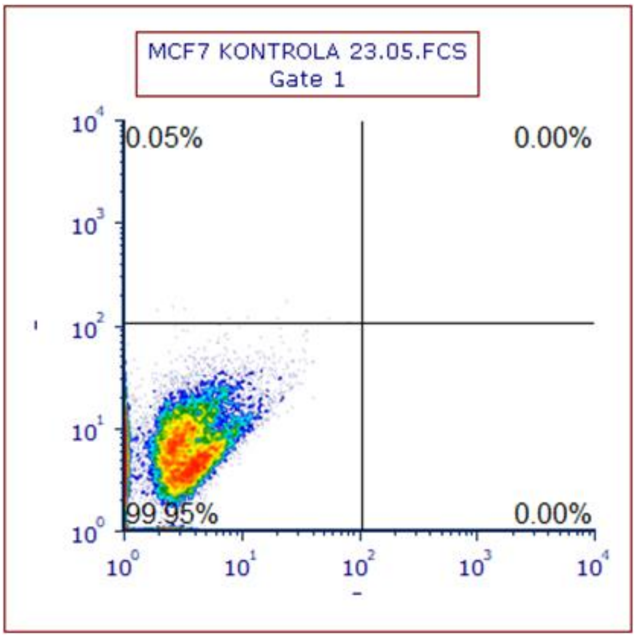

Figure S3. Representative cytogram for MCF-7 cells control.

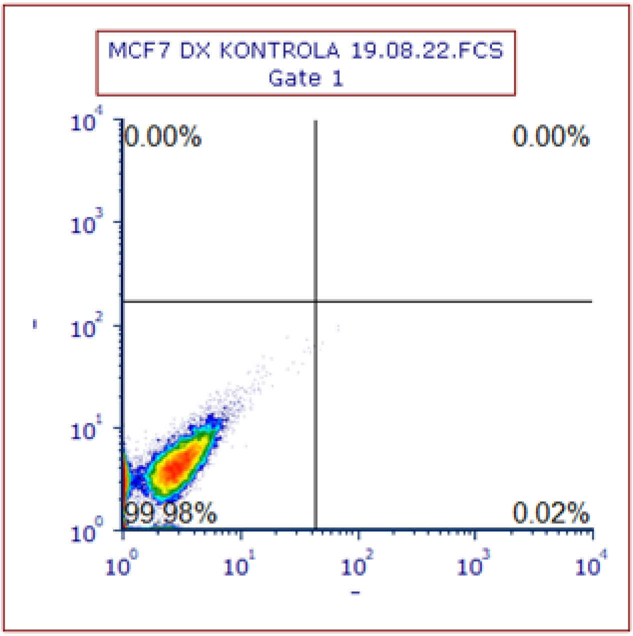

Figure S4. Representative cytogram for MCF-7/DOX cells control.
